# Seeing through the virtual reality: the effects of learning environment and task difficulty on difficulty perception, learning outcomes and mental models

**DOI:** 10.3389/fpsyg.2026.1772894

**Published:** 2026-02-17

**Authors:** Meixian Shan, Suying Du, Chenyu Shangguan, Mei Li

**Affiliations:** College of Education Science and Technology, Nanjing University of Posts and Telecommunications, Nanjing, China

**Keywords:** difficulty perception, learning outcome, mental model, task difficulty, VR

## Abstract

Virtual Reality (VR) offers learners highly immersive learning experiences through rich audiovisual stimuli and interactive environments, while traditional multimedia videos remain one of the most widely used instructional formats. However, existing research on the effects of these two learning environments (VR vs. multimedia videos) on learning outcomes, particularly knowledge transfer and mental models, has inconsistent conclusions, with task difficulty potentially playing a key role. Considering that different difficulty levels of learning tasks may trigger differentiated difficulty perceptions among learners in various environments, thereby influencing their learning outcomes and mental models, this study investigated how the interaction between learning environment and task difficulty affected learners’ difficulty perceptions, learning outcomes, and mental models. In the present study, we used a dual-task paradigm to investigate task difficulty processing under different learning environment conditions. This study included 92 college students and used a 2 (learning environment: VR vs. multimedia video) by 2 (task difficulty: high vs. low) between-subject factorial design. Results showed that: (1) In VR learning environment, students found high-difficulty tasks more challenging and low-difficulty tasks simpler; (2) VR learning environment enhanced knowledge transfer and mental model construction, particularly improving mental model development when tasks were highly difficult; (3) multimedia video learning environment was more conducive to knowledge retention, with better mental model development in the low-difficulty tasks condition. The study provides empirical evidence for the instructional design of immersive and traditional media environments, highlighting the importance of adaptive design strategies to optimize learning outcomes across varying cognitive demands.

## 1 Introduction

Virtual Reality (VR) creates an interactive 3D environment for learners through head-mounted displays and sensor systems, surpassing traditional 2D multimedia learning environments by simulating lifelike scenarios with enhanced sensory engagement (Luo et al., 2021). VR as a learning environment enables learners to improve learning outcomes, deepen understanding, and facilitate knowledge transfer (Krajcovc et al., 2022; Mills et al., 2024). However, research has found that the operational complexity and multimodal sensory engagement in a VR learning environment increased cognitive load and hindered learning outcomes (Poupard et al., 2025). Comparative studies show no significant difference in the knowledge transfer of the learning outcomes between VR and multimedia learning environments (Meyer et al., 2019), with some experiments showing superiority of multimedia groups (Parong and Mayer, 2018). The above researches imply inconsistent research findings on the impacts of VR learning environments on the learning outcomes, especially knowledge transfer, across different experimental tasks, suggesting a possible interaction effect between VR environments and task characteristics on learning outcomes.

Cognitive load theory provides a theoretical perspective for explaining the aforementioned contradictions. Sweller proposed that cognitive load can be categorized into three types: intrinsic cognitive load, which depends on the difficulty of the learning task itself (i.e., task difficulty in this study) and the learners’ prior knowledge level; extraneous cognitive load, which is directly influenced by the presentation of learning materials or content (i.e., learning environment in this study); and germane cognitive load (Paas and Van Merriënboer, 1994; Paas and Sweller, 2012; Sweller, 1988). Instructional designers need to tailor the difficulty of learning tasks to the characteristics of different learning environments to reduce intrinsic and extraneous cognitive load and promote learning outcomes and knowledge transfer (Ozerem and Akkoyunlu, 2015). Furthermore, learners’ difficulty perception during the learning process may moderate this process (Bandura et al., 1997; Belmont, 1987). Difficulty perception, a subjective evaluation of task difficulty from the learners’ perspective, is closely related to learning outcomes (Malmberg et al., 2022). The compatibility between the learning environment and task difficulty is crucial for optimizing cognitive load distribution. It is necessary to explore how task difficulty in a VR learning environment affects learners’ difficulty perception and learning outcomes.

Learning outcomes reflect the external application effectiveness of learners, whereas mental models represent their internal cognitive processes (van Merriënboer and Kirschner, 2012). Mental models are personal understanding and cognitive structures of the world, aiding in describing, explaining, and predicting complex systems (Johnson-Laird, 1986). When encountering new problems, individuals draw on chunks from their mental models, recombining and connecting them to form new knowledge and skill structures to adapt to novel situations (Vosniadou and Brewer, 1992). VR learning environments can create multisensory and highly interactive simulations that effectively develop mental models (Vogt et al., 2021). Most current research has focused on students’ academic performance and cognitive load (Zhang et al., 2024), motivation and attitudes (Klingenberg et al., 2020), cognition and emotions (Liu et al., 2025) within VR environments, with limited attention to VR learners’ mental models. This study investigated how different learning environments and task difficulties influenced learners’ difficulty perception, learning outcomes, and mental models. Examining learners’ perceived task difficulty clarified the subjective influence of VR learning environments, while learning outcomes and mental models are objective manifestations. Taken together, this study addressed the following questions:

RQ1: What are the effects of learning environment, task difficulty and interaction on learners’ difficulty perception?

RQ2: What are the effects of learning environment, task difficulty and interaction on learners’ learning outcomes?

RQ3: What are the effects of learning environment, task difficulty and interaction on learners’ mental models?

## 2 Literature review

### 2.1 VR and multimedia video learning environments

A learning environment is essential for teaching and learning, encompassing content, methods, and technical features. Multimedia learning theory suggests that video learning, which provides visual and auditory stimuli, enhances short-term memory and knowledge retention (Mayer, 2009). VR, a multi-channel learning environment, stimulates various senses, promoting active learning and knowledge construction. It adds a new dimension to multimedia learning environments, and the immersive learning experience in VR is essentially a form of multimedia learning (Mutlu-Bayraktar et al., 2019; Oje et al., 2025).

Research has found that learning environments affect learning outcomes through emotional paths represented by immersion and cognitive paths represented by cognitive load (Makransky and Petersen, 2019). Previous research has compared VR and multimedia video learning environments by setting the type of learning environment as the independent variable, and deeply analyzed their differences in learning outcomes and cognitive load (Çeken and Taşkın, 2022). In terms of learning outcomes, the Cognitive Affective Model of Immersive Learning (CAMIL) states that immersive learning environments positively impact learning achievement, including the learning outcomes of factual, declarative, and procedural knowledge, as well as knowledge transfer outcomes (Makransky and Petersen, 2021). Experimental research has found that compared with traditional teaching, the VR learning environment enables students to have in-depth experiences and perceptions, and improves their learning efficiency and outcomes (Huang et al., 2020). However, some studies have also found that the VR environment is affected by cognitive load and does not always enhance learning outcomes (Richards and Taylor, 2015).

A recent meta-analysis study reveals that an increasing number of researchers are paying attention to the impact of VR technology on cognitive load, but the conclusions are inconsistent (Wang et al., 2023). Studies have found that the VR learning environment can effectively reduce cognitive load and enhance learning outcomes in specific situations compared to the multimedia learning environment (Sari et al., 2024). Thus, VR environments are not always more effective than traditional multimedia environments in all learning tasks, and their applicability to different learning tasks, namely task difficulty, needs further exploration.

### 2.2 The role of task difficulty

Based on cognitive load theory, task difficulty is a key determinant of intrinsic cognitive load and how well learners acquire different types of knowledge (Sweller, 1988). Task difficulty is typically defined based on learners’ existing knowledge and experience. It is relatively objective and easy to operate, though its definition may vary across studies. In traditional learning environments, researchers have defined tasks of different difficulties in various ways, such as different types of subject knowledge (Gong et al., 2017), the production/solution steps (Gupta and Zheng, 2020) and knowledge complexity (Robinson, 2001). These studies consistently have found that learners with high-difficulty tasks experience higher cognitive loads and weaker learning outcomes.

Some studies have indicated that there are significant differences in difficulty perception when the learning process involves different types of tasks (Lee and List, 2021; Sadia Nawaz, 2022). Questions involving high cognitive demands, the use of various types of knowledge, and the need for learners to argue about scientific knowledge make learners feel that the tasks are more difficult (Sadia Nawaz, 2022). Some researchers have verified that conceptual tasks are easier to learn intuitively in multimedia video learning environments. When individuals master and apply various types of knowledge, they often need to perform more complex cognitive operations and deep thinking activities, which makes the tasks relatively more difficult (Wang et al., 2018). As for the VR learning environment, some researchers have found that VR helps individuals transform declarative knowledge into procedural knowledge (Li et al., 2019), which means that applying various types of knowledge requires more cognitive resources than elaborating on single knowledge concepts. Therefore, the difficulty of these tasks is higher for students.

In a VR learning environment, the complex role of task difficulty remains unclear. High-difficulty tasks require learners to allocate significant cognitive resources to handle internal loads. When combined with the high external load of VR itself, this can easily trigger cognitive overload, potentially exacerbating the decline in learning outcomes (Parong and Mayer, 2021). Conversely, some research indicates that learners in VR environments with higher external cognitive loads may exhibit stronger comprehension abilities (Skulmowski and Rey, 2020). Interactive learning in VR might stimulate more active thought processes, enabling learners to use their acquired knowledge more flexibly when facing creative high-difficulty tasks, thereby enhancing the quality of task completion (Yu et al., 2023).

### 2.3 Difficulty perception

In Efklides’ metacognitive, motivational, and affective model, difficulty perception is a metacognitive experience, i.e., learners’ subjective feelings and evaluations of task difficulty (Efklides and Schwartz, 2024). Studies indicate that multimedia learning environments can significantly reduce learners’ difficulty perception, thereby improving learning outcomes (Chiou and Lin, 2025; Plass et al., 2014; Um et al., 2012). Is there a similar effect in a VR environment? For more detailed classification, the impact of task difficulty should also be taken into account. Based on relatively low-difficulty tasks, does the VR environment reduce the difficulty perception to some extent compared to the multimedia video environment due to its immersion? But as the task difficulty increases, does students’ difficulty perception also rise in a VR learning environment? The manifestation of difficulty perception brought by different task difficulties in VR has not yet been studied. Furthermore, high-difficulty tasks can enhance learners’ performance, showing that appropriate difficulty can motivate learners and boost learning outcomes (Aljamal et al., 2019). However, some studies reveal that learners with high difficulty perception, who consider academic tasks more difficult, tend to have poorer learning outcomes (Power et al., 2020).

### 2.4 Learning outcomes

In VR-based empirical studies, learning outcomes are usually divided into knowledge retention and knowledge transfer (Moreno and Mayer, 2002). Knowledge retention evaluates students’ ability to remember and retain learned content, while knowledge transfer measures their ability to apply knowledge in new situations. The study has pointed out in their meta-analysis study that VR advantage lies in promoting knowledge retention but has less positive impact on knowledge transfer (Coban et al., 2022). The research has found that VR significantly enhances knowledge transfer compared to a video learning environment (Makransky et al., 2019). However, in another experiment conducted by the same research team, the knowledge transfer effects of VR learners were lower than those in the video learning environment (Makransky et al., 2021).

The impact of VR learning environments on learning outcomes varies because of different task conditions. Researchers have discovered that learners’ inefficient cognitive resource distribution in VR environments means knowledge transfer efficiency in the relatively low-difficulty tasks is not significantly higher than in video learning (Gao et al., 2023). Nevertheless, VR leads to better knowledge transfer in the relatively high-difficulty tasks. The study has found that the VR learning environment boosts knowledge transfer in tasks focusing on application but does not significantly affect tasks focusing on concept learning (Zhang et al., 2024). Research illustrates VR learning can contextualize learning tasks, especially high-difficulty tasks requiring knowledge transfer (Liu et al., 2021).

### 2.5 Mental model

Multimedia video learning environments can promote the formation and improvement of learners’ mental models, enabling them to better apply knowledge in practical situations (Hegarty, 2014; Riemer and Schrader, 2019). VR offers a more comprehensive technical medium than text and image multimedia environments. On the one hand, VR tools allow students to build advanced mental models by viewing dynamic angles and interacting with learning environment components through 3D simulations (Chen et al., 2015). Many studies have explored how different learning environments impact students’ mental models. Within these, 3D simulations enhance and complete students’ mental models compared to 2D representations (Wu and Chiang, 2013). On the other hand, one study reported that 2D animations and graphics can help students construct new-concept mental models (Mayer, 2002). Thus, existing literature presents conflicting conclusions on whether dynamic learning environments are better than static text and image ones for mental model construction, and few studies have compared them with a VR learning environment. A study has concluded that the VR learning environment can improve primary school students’ scientific knowledge transfer and mental models (Li et al., 2023a; Li et al., 2023b; Li et al., 2023c).

Task difficulty also impacts mental model construction. In mental model theory, when tasks or problems are complex, individuals need to build more detailed and complex mental models to process information (Johnson-Laird, 2001). In other words, when task difficulty is high, task difficulty increases working memory demands, requiring learners to build more complex mental models, thus facilitating better development of learning outcomes (Zhang, 2012). Currently, research on how mental models promote knowledge retention and transfer is limited, with few empirical studies considering the factor of task difficulty in learning. Traditional methods for assessing mental models, such as verbal reports and concept mapping, have been used in prior studies (Atasoy et al., 2020; Li et al., 2023a; Li et al., 2023b; Li et al., 2023c). Therefore, this study used concept mapping and verbal descriptions to assess students’ mental models.

To sum up, existing research has made certain progress in aspects such as comparing VR and multimedia video learning environments, the impact of task difficulty on learning outcomes, and the role of difficulty perception and mental models. However, the following key issues still need to be explored in depth: (1) The insufficiency of interaction effect research. Although the cognitive load theory emphasizes the importance of adapting the learning environment to task difficulty, relevant empirical research is still lacking. The inconsistency of the existing research conclusions may be partly due to neglecting the interaction effect between this crucial effect. (2) The weakness of mental model research in the VR environment. Although VR is believed to promote spatial understanding and model construction, compared with other common indicators, the VR learning environment lacks empirical evidence for learners’ mental models and related dimensions under different task difficulties. It is still unclear whether and under what task difficulty VR can better promote the construction of complex mental models. (3) The lack of empirical evidence on the difficulty perception. As a core variable of learners’ subjective experience, difficulty perception is affected by task difficulty, but the influence of the learning environment on it remains unclear.

In summary, existing studies have drawn inconsistent conclusions regarding the impact of VR on learning outcomes, primarily because the learning tasks and their corresponding task difficulty levels vary across studies, leading to differences in learners’ difficulty perception. Moreover, due to the immersive nature of VR learning environments, which distinguishes them from multimedia video learning environments, difficulty perception becomes even more elusive. Learning outcomes reflect the external manifestation of knowledge acquisition and application, while mental models, as the internal cognitive structures constructed by learners, form a complementary relationship between external behavioral performance and internal cognitive mechanisms. Therefore, this study takes difficulty perception, learning outcomes, and mental models as dependent variables to systematically explore how the interaction between different learning environments and task difficulties simultaneously affects learners’ subjective difficulty perception, external learning effectiveness, and internal cognitive structures, thereby providing more comprehensive theoretical support and practical implications for the instructional adaptation design of VR and traditional media.

This study conducted a 2 × 2 experiment to explore how different learning environments (VR vs. multimedia video) and task difficulties (high vs. low) affected learners’ difficulty perception, learning outcomes (knowledge retention and transfer), and mental model. Based on the research questions and literature review, the following research hypotheses are proposed:

*H1*: Learning Environment, Task Difficulty, and their interaction affect learners’ difficulty perception.

*H1a*: Learners’ difficulty perception is higher in the High-difficulty Task than in the Low-difficulty Task.

*H1b*: Learners’ difficulty perception is higher in the VR Learning Environment than in the Video Learning Environment.

*H1c*: Compared with the Video Learning Environment, the increase in learners’ difficulty perception is more pronounced in the VR Learning Environment under the High-difficulty Task.

*H2*: Learning Environment, Task Difficulty, and their interaction affect learners’ learning outcomes.

*H2a*: Learners’ learning outcomes are better in the High-difficulty Task than in the Low-difficulty Task.

*H2b*: Learners’ learning outcomes are better in the VR Learning Environment than in the Video Learning Environment.

*H2c*: Compared with the Video Learning Environment, the VR Learning Environment exerts a more prominent promoting effect on learners’ learning outcomes under the High-difficulty Task.

*H3*: Learning Environment, Task Difficulty, and their interaction affect learners’ mental models.

*H3a*: Learners’ performance of mental models is better in the High-difficulty Task than in the Low-difficulty Task.

*H3b*: Learners’ performance of mental models is better in the VR Learning Environment than in the Video Learning Environment.

*H3c*: Compared with the Video Learning Environment, the VR Learning Environment exerts a more prominent promoting effect on learners’ performance of mental models under the High-difficulty Task.

## 3 Research design

### 3.1 Participants

An *a priori* sample size estimation for the study was performed using G*Power 3.1.9.7 software. A large effect size (*f* = 0.4) for the two-way between-subjects analysis was adopted as the effect size estimate for this study, and in accordance with the study design, a total of 52 participants were required to achieve a statistical power (1 − *β* = 0.80, *α* = 0.05). This study recruited 92 participants through QQ, WeChat, and posters, with an age range of 18–26 years old and a mean age of 21.63 years old, of which 35 were male, accounting for 38%. Because the learning content was related to biology, none of the participants majored in biology, but all had studied biology in high school and achieved good or excellent grades in biology in college or high school exams. All participants gave their informed consent to the experiment. Before the experiment, participants were randomly assigned to different groups through a 1–4 random number generator and finally were divided into four groups: video low-difficulty task group (*n* = 23), video high-difficulty task group (*n* = 23), VR low-difficulty task group (*n* = 23), and VR high-difficulty task group (*n* = 23).

### 3.2 Research materials

Researchers have found that VR technology in STEM education (science, technology, engineering, and mathematics) improves academic achievement more than traditional teaching (Chen and Chu, 2024; Tai and Hong, 2025). Thus, Biology was chosen as the learning content. The experiment required two learning environments (video and VR), and the learning content chosen for the study was “The Body VR: Journey Inside a Cell”, a popular science resource on the STEAM platform. It allows learners to enter the human body in the form of microscopic cells, flow with blood cells, enter damaged cells to observe structures, and work with immune cells to fight viruses. This learning content has been used by previous studies (Meyer et al., 2019; Parong and Mayer, 2021; Zhang et al., 2023). The VR learning environment consisted of HTC VIVE Pro Eye glasses paired with an interactive joystick, which was worn by the subject with a head-mounted display to navigate through cells in an immersive manner. Specifically, learners were situated in a fixed cell cabin and traveled along a preset track during the VR tour, and the VR experience featured fixed sessions and scripted operation, thus ensuring the consistency of core learning content and the controllability of the experience process. For interactions, learners could only launch cells freely in place within a range of approximately 2–4 square meters, and turn their heads 360 degrees to observe different types of cells and the internal structures of cells. Handles enabled two interactions: touching cells to make them rotate or move, and long-pressing to launch cells into virtual environments. On the other hand, the video learning environment was presented by the Legion Y7000 computer, and was the same footage of the VR environment recorded in a fixed perspective that displays all of the knowledge point information. Learners watched with headphones, unable to control the playback. Except for the presentation method, both environments had the same content, knowledge points, and 10-min and 35-s durations. Neither the VR nor the video learning environment allowed learners to pause operations.

According to the literature review mentioned above, the task difficulty in this study was developed by offering learners learning tasks of different difficulties. The experimental designer drew on a previous study (Meyer et al., 2019). A college biology teacher checked the tasks for completeness, rationality, logic, and difficulty. After the prior knowledge test, refinements and optimizations were made. The low-difficulty task focused on conceptual understanding. It had three questions, such as “Briefly describe how the immune system fights off viral invasions, including the roles of antibodies and white blood cells.” Students needed to understand the immune system’s structure and functions and present the knowledge in a well-organized way. The high-difficulty task targeted cognitive strategies and thinking transformation. It also had three questions, such as “In a lab, you are looking at a blood sample where the infection has spread more than expected. What could be the reason?” Students should use their acquired knowledge to analyze a real-world problem and find possible causes. Both tasks were designed with 5 scoring points, 2 points. Upon completion of the experiment, two raters independently scored learners’ task completion performance. A reliability test showed the correlation coefficient of the scoring results reached 0.92, indicating a high level of inter-rater consistency, which was defined as *task completion*.

### 3.3 Measures

*Prior knowledge* was measured by the basic knowledge of biological cells to understand students’ familiarity with the learning content, to avoid interference with the results of the experiment due to differences in the subjects’ level of knowledge, and the results of the test were not be fed back to the subjects. The prior knowledge test questions were mainly compiled by the biology professional teaching staff, while referring to the learning content as well as the refinement and optimization of the test questions through the prior knowledge test, which contained a total of 10 true or false questions (1 point each, 10 points total). Specific example questions are shown in Table 1.

**Table 1:** Sample test questions.

Test type	Question types	Example
Prior knowledge test	True or false questions	Macrophages are a type of protein.
Retention test	Multiple-choice questions	What is another name for mitochondria? Cells are power stations. Cell controller. The source of power for cells. DNA memorizer.
Transfer test	Short-answer questions	At the end of the simulation of a human blood trip, the cell loses its fight against the virus, and the virus invades the nucleus of the cell. Guess with the help of which substance the virus crosses the cell membrane and enters the interior of the cell, and how does this substance help it to succeed in entering the nucleus of the cell?

*Difficulty perception* is generally measured through subjective ratings, asking participants about their perceived task difficulty, ranging from very simple to very difficult. In this study, participants rated their difficulty perception on a percentage scale after completing the learning task, where 0 indicates no difficulty at all and 100 indicates very high difficulty. The higher the score, the more difficult the learning task was perceived by the learners. There has been no unified measurement scale for difficulty perception in previous studies, with scales commonly using 1–5, 1–7, or 1–9 Likert scoring. Considering the group characteristics of the research participants (undergraduates and postgraduates), this study adopted a 1–100 continuous scoring method to assess difficulty perception, referring to the relevant research (Huang et al., 2023). This approach was intended to achieve a refined quantitative evaluation of difficulty perception and improve the accuracy of the assessment results.

*Learning outcomes* are evaluated via retention and transfer tests. Based on tests from previous studies (Meyer et al., 2019; Zhang et al., 2023), and modified by a biology teacher, the test included 10 multiple-choice questions on knowledge retention (1 point each, 10 points total) and 2 short-answer questions on knowledge transfer (4 and 8 points, 12 points total), as shown in Table 1. To ensure the effectiveness of students’ answers, the test session provided students with the same and sufficient time for answering the questions. After the completion of the experiment, two raters were asked to score and test the scoring results. The correlation coefficient was 0.83, the scoring consistency was good, and the average was taken to get the knowledge retention and transfer scores.

*Mental model* was compiled with reference to the study of a previous study (Li et al., 2023a; Li et al., 2023b; Li et al., 2023c) It consisted of three main dimensions: (1) written explanation, which required describing the characteristics of the cell (total score of 4); (2) drawing explanation, which required drawing the cell and its characteristics (total score of 10); and (3) detail explanation, which required explaining the steps of drawing (total score of 6). After the experiment, two raters scored the mental model, and the rating results were tested. The correlation coefficient was 0.88, indicating high consistency. The average score was taken as the score for each dimension, and the total score of the mental model was the sum of the scores of the three dimensions, as shown in Table 2.

**Table 2:** Mental model test.

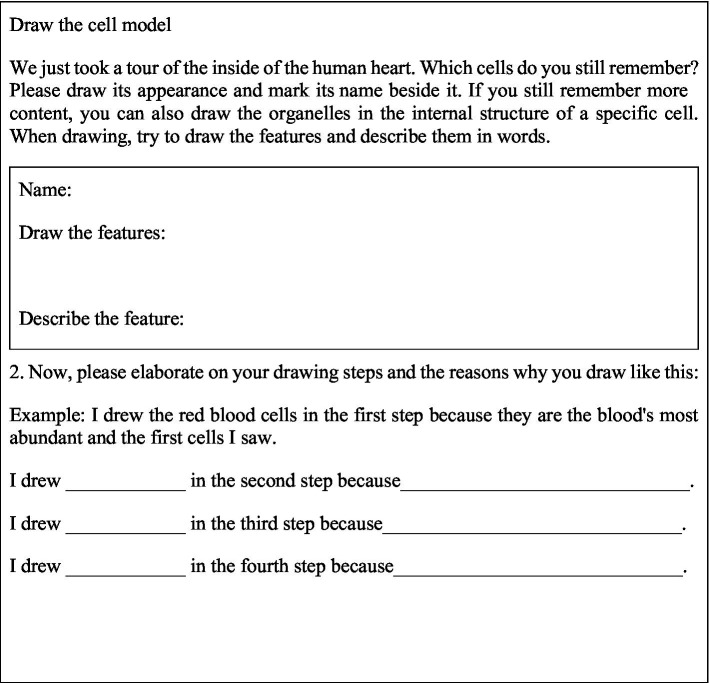

### 3.4 Procedure

The experimental process was divided into four steps: (1) Experimental subjects signed an informed consent form. The experiment instructor guided participants in using VR and conducted VR adaptation training, recording any discomfort experienced. This ensured that each participant was objective and comfortable. (2) The subjects in each group filled in the basic information, a prior knowledge test, and read the learning task. (3) The subjects in each group wore the device and learned the designated learning content. (4) After the subjects finished learning, they completed the difficulty perception score, learning performance test, and mental model.

## 4 Results

### 4.1 Manipulation check

A one-way analysis of variance (ANOVA) was conducted on the prior knowledge test results, and no significant differences were found among the samples of different groups, as shown in Table 3. This indicated that the participants in each group had consistent initial levels with no innate inter-group differences (*F* = 0.08, *p* = 0.969), thus ensuring the validity and reliability of the teaching intervention.

**Table 3:** Descriptive statistics for prior knowledge test and task completion (*M ± SD*).

Variables	Low-difficulty task video group (*n* = 23)	High-difficulty task video group (*n* = 23)	Low-difficulty task VR group (*n* = 23)	High-difficulty task VR group (*n* = 23)
Prior knowledge test	8.52 ± 1.16	8.48 ± 1.44	8.35 ± 1.23	8.43 ± 1.08
Task completion	7.87 ± 0.63	5.35 ± 2.37	7.09 ± 1.86	5.96 ± 3.07

Since the preset task difficulty was relatively subjective to the researchers themselves, it still needed to be based on the real difficulty perception of the learners. Students rated the difficulty of the tasks they received before learning. The analysis showed a significant difference (*t* = −5.95, *p* = 0.000, *Cohen’s d* = 1.240, large effect size), and the mean difficulty perception of the low-difficulty task (42.74) was significantly lower than the mean of the high-difficulty task (68.11). This indicated students’ difficulty perception difference between the two kinds of difficulty tasks, supporting the task difficulty classification with objective data. There was also a significant difference in task completion (*t* = 4.03, *p* = 0.000, *Cohen’s d* = 0.839, large effect size). The mean of the low-difficulty tasks (7.48) was significantly higher than the mean of the high-difficulty tasks (5.65), as shown in Table 4. Therefore, it demonstrated that the operational difficulty of this research task was valid.

**Table 4:** Differences in difficulty perception and task completion by task difficulty (*M ± SD*).

Task difficulty groups	Low-difficulty tasks (*n* = 46)	High-difficulty tasks (*n* = 46)	*t*	*d*
Difficulty perception	42.74 ± 20.59	68.11 ± 20.32	5.95**	1.240
Task completion	7.48 ± 1.43	5.65 ± 2.73	4.03**	0.839

*p < 0.05, **p < 0.01.

### 4.2 Descriptive statistics

To examine the effects of different learning environments and different task difficulties on learners’ difficulty perceptions, learning performance and mental model, the present study used the software SPSS 25.0 and the R programming language to conduct a two-way factor ANOVA. The descriptive statistics of the subjects in the experiment are shown in Table 5.

**Table 5:** Descriptive statistics for each variable under the four groups of experiments.

Variables		Low-difficulty task video group (*n* = 23)		High-difficulty task video group (*n* = 23)		Low-difficulty task VR group (*n* = 23)		High-difficulty task VR group (*n* = 23)	
		*M*	*SD*	*M*	*SD*	*M*	*SD*	*M*	*SD*
Difficulty perception		48.57	23.29	61.09	24.26	36.91	15.93	75.13	12.37
Knowledge retention		7.17	1.37	6.91	1.51	6.43	1.44	6.65	1.19
Knowledge transfer		6.78	2.54	7.22	1.93	8.13	3.31	8.17	2.73
Mental model	Written explanation	2.00	1.57	2.04	2.10	2.52	1.38	1.35	1.64
	Drawing explanation	7.09	2.45	5.39	2.34	7.30	3.01	7.91	2.78
	Detail explanation	5.13	1.87	5.26	2.05	4.78	1.91	5.39	1.83

Pearson correlation coefficients were computed to examine the linear associations between the two independent variables and all dependent variables. Knowledge transfer (*r* = 0.22, *p* = 0.040) and drawing explanation (*r* = 0.25, *p* = 0.018) were significantly positively correlated with learning environment grouping, whereas difficulty perception was strongly positively correlated with task difficulty grouping (*r* = 0.53, *p* < 0.001). For mental models, detail explanation was significantly correlated with written explanation (*r* = 0.25, *p* = 0.014) and highly significantly correlated with drawing explanation (*r* = 0.28, *p* = 0.007). No other significant correlations were observed. These results offered valuable preliminary insights into the variable interrelationships.

### 4.3 The effect of learning environment and task difficulty on difficulty perception

**Figure 1:**
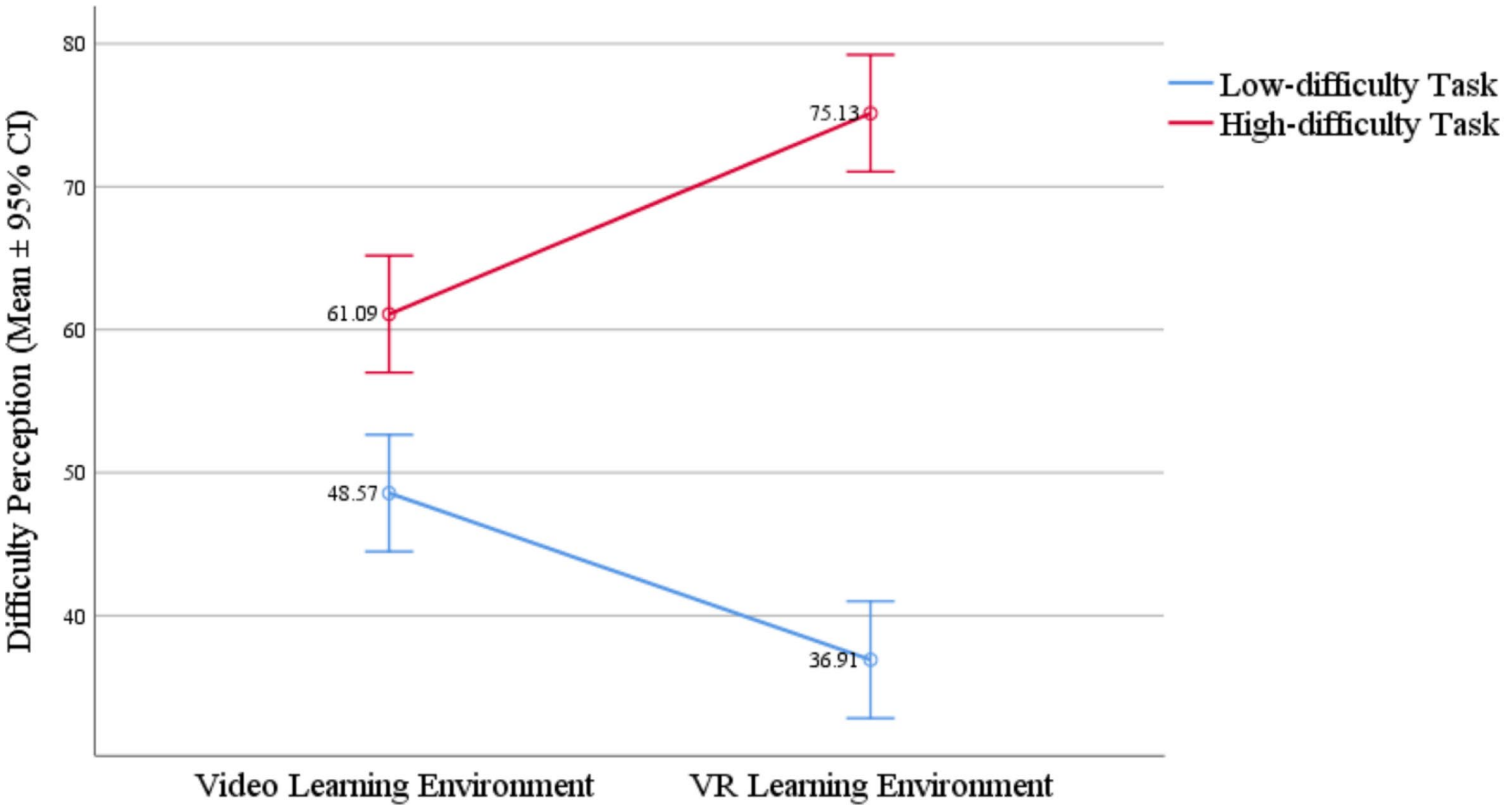
Interaction between learning environment and task difficulty on difficulty perception.

A two-factor ANOVA was conducted with learning environment and task difficulty groups as independent variables and difficulty perception as the dependent variable, as shown in Figure 1. The results indicated a significant main effect of task difficulty (*F* = 38.51, *p* = 0.000, *η_p_^2^* = 0.304, large effect size), showing that task difficulty predictably influenced difficulty perception, with higher task difficulty associated with stronger difficulty perception among learners. H1a was supported. The learning environment had no significant effect on difficulty perception (*F* = 0.086, *p* = 0.771). H1b was not supported. Moreover, the interaction between learning environment and task difficulty groups was significant (*F* = 9.876, *p* = 0.002, *η_p_^2^* = 0.101, medium effect size).

Further simple effects analysis and comparisons of means and standard deviations indicated that in the low-difficulty task, the difficulty perception of the VR group was 11.65 points lower significantly than that of the video group [*F*(1, 88) = 4.06, *p* = 0.047, *η_p_^2^* = 0.044, small effect size]. In the high-difficulty task, the difficulty perception of the VR group was 14.04 points higher significantly than that of the video group [*F*(1, 88) = 5.90, *p* = 0.017, *η_p_^2^* = 0.063, medium effect size]. Therefore, the impact of the learning environment on difficulty perception varied depending on the task difficulty: in low-difficulty tasks, the VR environment reduced difficulty perception; in high-difficulty tasks, the VR environment increased difficulty perception.

Additionally, in the video environment, the difficulty perception of the high-difficulty task was 12.52 points higher significantly than that of the low-difficulty task [*F*(1, 88) = 4.69, *p* = 0.033, *η_p_^2^* = 0.051, small effect size]. In the VR environment, the difficulty perception of the high-difficulty task was 38.22 points higher significantly than that of the low-difficulty task [*F*(1, 88) = 43.70, *p* = 0.001, *η_p_^2^* = 0.332, large effect size]. Therefore, task difficulty had a significant effect on difficulty perception in both environments, but the effect size of the difference between high and low difficulty tasks in the VR environment far exceeded that in the video environment. This indicated that the VR environment was more sensitive to changes in difficulty perception. H1c was supported. H1 was partially supported.

### 4.4 The effect of learning environment and task difficulty on learning outcomes

A two-factor ANOVA of learning outcomes showed that transfer scores were significant in the learning environment groups [*F*(1, 88) = 4.28, *p* = 0.041, *η_p_^2^* = 0.046]. The VR group had higher knowledge transfer scores than the video group, indicating VR was more effective for knowledge transfer. Knowledge retention scores showed marginal significance [*F*(1, 88) = 3.01, *p* = 0.086], with the video group having higher retention scores, suggesting video learning had a slight short-term memory and knowledge retention advantage. H2b was partially supported. Task difficulty groups had no significant impact on knowledge transfer [*F*(1, 88) = 0.18, *p* = 0.669] and knowledge retention [*F*(1, 88) = 0.01, *p* = 0.940]. H2a was not supported. There was also no interaction between learning environment and task difficulty groups on knowledge transfer [*F*(1, 88) = 0.12, *p* = 0.726] and knowledge retention [*F*(1, 88) = 0.69, *p* = 0.409]. H2c was not supported. Thus, compared to multimedia learning environments, VR was more effective for knowledge transfer. H2 was partially supported.

### 4.5 The effect of learning environment and task difficulty on mental model

A two-factor ANOVA on mental model scores showed that in the learning environment groups, drawing explanation differed significantly [*F*(1, 88) = 6.07, *p* = 0.016, *η_p_^2^* = 0.064]. The VR group had a higher mean (7.61) than the video group (6.24), indicating VR learning benefited spatial or 3D mental model construction. No significant differences were found in written explanations [*F*(1, 88) = 0.06, *p* = 0.806] and detail explanations [*F*(1, 88) = 0.07, *p* = 0.786] between the video and VR environment. H3b was not partially supported.

A two-factor ANOVA on mental model scores showed that in the task difficulty groups, no significant differences were found in drawing explanation [*F*(1, 88) = 0.96, *p* = 0.331], written explanations [*F*(1, 88) = 2.57, *p* = 0.113] and detail explanations [*F*(1, 88) = 0.86, *p* = 0.357] between the low-difficulty and the high-difficulty tasks. H3a was not supported.

**Figure 2:**
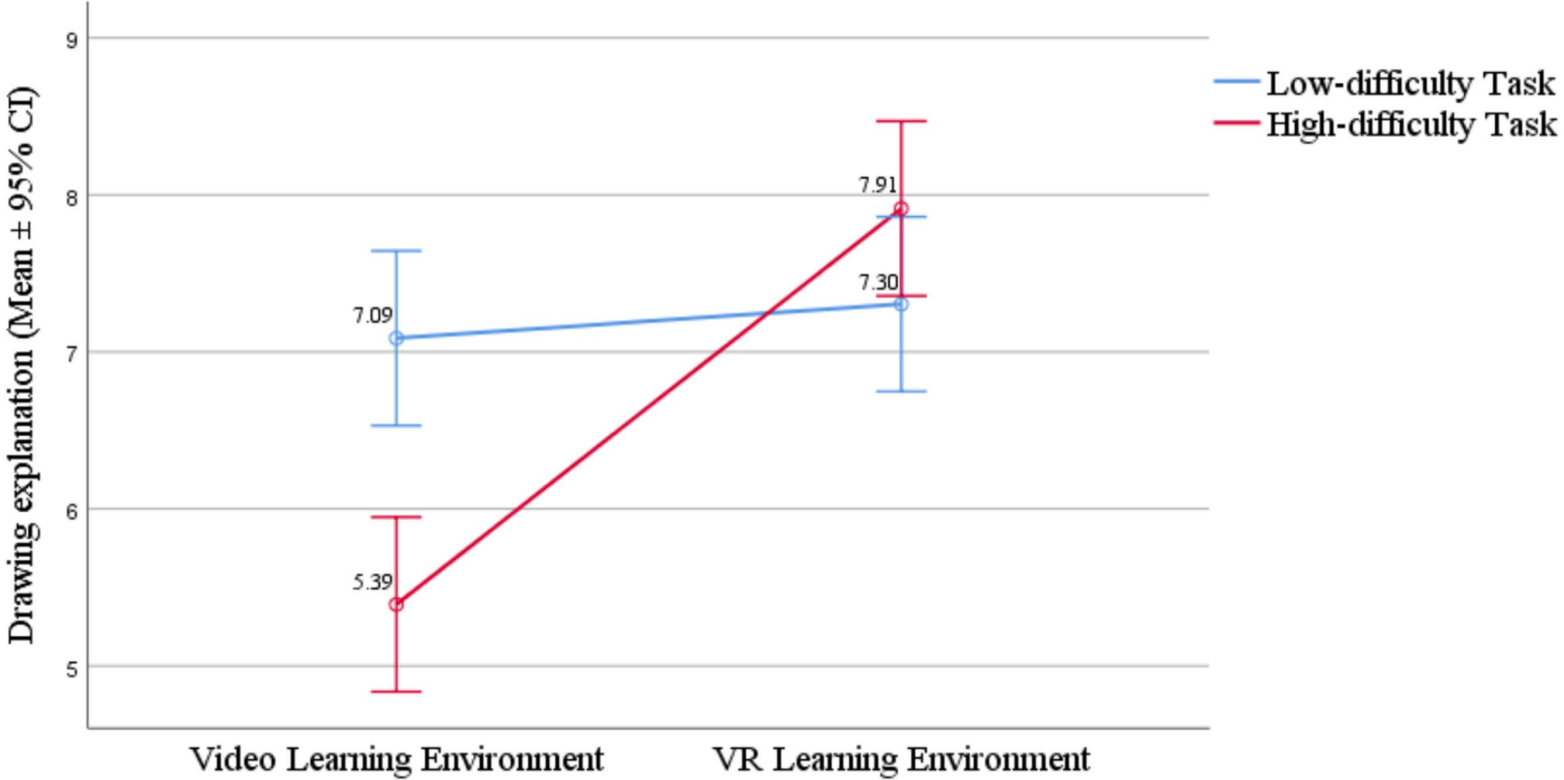
Interaction between learning environment and task difficulty on drawing explanation.

A two-factor ANOVA on mental model scores revealed a significant interaction between learning environment and task difficulty in the drawing explanation dimension [*F*(1, 88) = 4.30, *p* = 0.041, *η_p_^2^* = 0.047] as shown in Figure 2. Further simple effects analysis and comparisons of means and standard deviations revealed that in the low-difficulty task, the VR group scored 0.22 points higher in drawing explanation than the video group, with no significant difference [*F*(1, 88) = 0.08, *p* = 0.783]. In the high-difficulty task, the VR group scored 2.52 points higher significantly in drawing explanation than the video group [*F*(1, 88) = 10.28, *p* = 0.002, *η_p_^2^* = 0.105, medium effect size]. Thus, the advantage of the VR environment in fostering mental models was evident only in high-difficulty tasks. H3c was supported.

Additionally, in the video environment, the high-difficulty task scored 1.70 points lower significantly in drawing explanation than the low-difficulty task [*F*(1, 88) = 4.65, *p* = 0.034, *η_p_^2^* = 0.050, small effect size]. In the VR environment, the high-difficulty task scored 0.61 points higher in drawing explanation than the low-difficulty task, with no significant difference [*F*(1, 88) = 0.60, *p* = 0.441]. Therefore, the low-difficulty task was more conducive to the development of drawing explanation in learners’ mental models within the video environment.

There was also no interaction between the low-difficulty and the high-difficulty task group on written explanations [*F*(1, 88) = 2.98, *p* = 0.088] and detail explanations [*F*(1, 88) = 0.36, *p* = 0.551]. H3a was not supported. Overall, VR environment learners showed better mental model development than video environment learners, especially under high-difficulty tasks. Thus, the combination of VR environment and high-difficulty tasks was most effective for mental model development, while the multimedia environment with low-difficulty tasks also supported good mental model development. H3 was partially supported.

## 5 Research discussion

### 5.1 The effect of learning environments and task difficulty on difficulty perception

In response to research question 1, this study revealed that the VR learning environment does not simply increase or decrease difficulty perception, but magnifies the perception differences brought about by task difficulty itself. Specifically manifested as follows: In low-difficulty tasks, the VR environment makes learners feel that the tasks are simpler; In high-difficulty tasks, the VR environment makes learners feel that the tasks are more difficult. This reveals the complex influence of the immersive characteristics of VR on the subjective experience of learners, and its direction of action is highly dependent on the objective task difficulty itself.

This study also reveals that despite the increased cognitive load in a VR learning environment, which heightens students’ perceived task difficulty, it still facilitates knowledge transfer and mental model development.

The immersive nature of a VR learning environment may lead to distraction or misallocation of resources when tasks are perceived to be easier, thus reducing the quality of students’ learning input. Prior studies suggest that moderately increasing the subjective difficulty perception of learning content can benefit learning (Lehmann et al., 2016; Xie et al., 2016). However, as students’ subjective difficulty perception rises, the high cognitive load inherent in VR environments did not impede learning as predicted by traditional cognitive load theory. The high level of difficulty perception may allow learners to focus their learning objectives and cognitive resources primarily on task processing, resulting in a self-regulated learning process, coupled with a VR environment that allows students to explore and interact more actively, thus facilitating understanding and interpretation of details. However, can the excessive difficulty perception also promote the learning effect? Some studies have found that learners’ difficulty perception systemically increases, which eventually leads to learners’ withdrawal from self-regulated learning (Cleary et al., 2015; Malmberg et al., 2022; Sadia Nawaz, 2022), but the impact on learning effect is not clear yet. This may be explored in future research.

### 5.2 The effect of VR learning environment on learning outcomes

In response to research question 2, this study found that the VR environment showed better knowledge transfer. The immersive VR experience allows students to learn and practice in a more authentic environment, which facilitates knowledge transfer and application. The video group had relatively better knowledge retention. The intuitive and concise nature of video materials helps students quickly absorb and memorize knowledge points. The VR group outperformed the video group in drawing explanation and overall mental model, indicating that VR helps students build more comprehensive and three-dimensional mental models. Previous studies have also demonstrated that the VR learning environment can promote knowledge transfer, while the video learning environment aids knowledge retention. For instance, the study found that virtual experiment systems assist in the transfer of scientific concepts (Li et al., 2019). Experimental results show that video-based multimedia material generates the best learning performance for verbal style learners (Chen and Sun, 2012). A study revealed that learners’ verbal and visual mental models develop better in VR environments (Li et al., 2023a; Li et al., 2023b; Li et al., 2023c).

The lack of significant effects from task difficulty groups and the interaction between learning environment and task difficulty groups on academic achievement might be explained by the impact of task difficulty on learners’ motivation regulation strategies (Wolters, 1998). Motivation regulation, a key part of self-regulated learning, involves learners strategically inspiring, maintaining, and enhancing their motivation to initiate or complete learning tasks (Wolters, 2003). In this study, high-difficulty tasks may have prompted learners to employ more motivation regulation strategies. Research has demonstrated that students use self-regulation in video learning, which requires them to actively construct and test mental models (Brame, 2016). Compared to multimedia video environments, VR learning environments allow learners to interact with elements (e.g., adjusting their view to avoid dense cells, actively emitting cells to eliminate viruses), creating a highly engaging learning environment. Given that participants had limited VR experience, the immersive educational game may have sparked their curiosity and focus, enhancing their interest. Thus, learners might have sustained greater interest and enthusiasm for the learning tasks, increasing their motivation to overcome task difficulty and cognitive load (Sweller, 1994). This could have led to no significant results for task difficulty and interaction effects.

Future research can further explore the impacts of learners’ motivation, emotions, and cognitive load through empirical studies. In teaching practice, for quickly mastering and memorizing knowledge points, video learning is more suitable. For deeply understanding and applying knowledge, if conditions permit, VR learning is a better choice. Schools without extensive VR facilities can use videos recorded in VR form as an alternative due to their stronger sense of immersion.

### 5.3 The effect of learning environments and task difficulty on mental model

In response to research question 3, this study found that the VR learning environment could enhance learners’ mental models, particularly in drawing explanation during high-difficulty tasks, indicating that VR boosted students’ spatial perception and visualization abilities. In contrast, in the video learning environment, mental models based on drawing explanation in low-difficulty tasks developed more effectively. These results are comparable to the findings of a previous study (Zhang et al., 2024). A literature review also suggests that VR is most commonly used for teaching applied learning tasks (Radianti et al., 2020). The study discovered that under certain conditions, VR could significantly make learners more competent in applied tasks (Li et al., 2023a; Li et al., 2023b; Li et al., 2023c). The measurement of drawing and written explanations in the mental model bears some resemblance to the graphic and explanation strategies in generative learning strategies. A VR learning environment provides learners with authentic contexts. The study, through an eye-tracking experiment, further found that in the VR learning environment, graphic strategies could improve learners’ information selection processes, leading to more comprehensive development of mental models; while in the video learning environment, graphic strategies made learners’ mental models more coherent (Fiorella et al., 2020). The synchronous presentation of visualized knowledge graphs and instructional content in videos aligns with the dual-channel assumption (visual and auditory channels) in multimedia learning theory, facilitating effective information encoding and storage, and thereby enhancing knowledge retention (Brame, 2016).

In a VR learning environment, teachers can integrate videos, images, texts, and other media to offer diverse information channels, boosting mental model development. They should also incorporate suitable generative learning strategies. This helps students grasp complex content and form coherent mental representations.

### 5.4 Limitations

The study had the following limitations: (1) The combinations of VR environment with high-difficulty tasks and multimedia environment with low-difficulty tasks were found to be optimal for learning outcomes, but the categorization was broad and could be further refined. (2) The research was set in a biological context and concluded that VR promoted knowledge transfer and mental models. However, the impact of the VR learning environment and task difficulty on other subjects is also worth exploring to enrich VR learning environment designs. (3) This study did not further explore the relationship between difficulty perception, learning outcome, and mental models. Previous studies have shown that difficulty perception can affect students’ learning outcomes. But in this study, students’ learning outcomes were simultaneously influenced by the learning environment and task difficulty, making it difficult to distinguish whether difficulty perception also had an impact. When the author analyzed difficulty perception as a mediating variable, the effect was not significant. Future research can continue to explore the role of difficulty perception in VR learning environments.

## 6 Conclusion

This study conducted a 2 × 2 experiment to explore how different learning environments (VR vs. multimedia video) and task difficulties (high vs. low) affect learners’ difficulty perception, learning outcomes (knowledge retention and transfer), and mental model. The findings of this study provided important empirical basis and specific inspirations for educational practice: (1) Environmental selection and task design need to be adapted. (2) Select a plan based on the learning objectives. When the learning goals focus on knowledge transfer and application and the deeply construction of mental models, VR is a better choice; When focusing on the knowledge retention of basic concepts, well-designed multimedia videos may be more efficient and economical. (3) Pay attention to the subjective experience of learners (difficulty perception). In VR instructional design, it is crucial to monitor and guide learners’ perception of difficulty. Teachers should help learners stay in the “optimal zone” of difficulty perception by providing appropriate brackets or designing tasks by taking advantage of the interactive features of the VR environment. (4) Complementarity and integration of VR and video environments. Recognizing the advantages of both environments, VR and video can be integrated and used in course design. For example, first introduce the basic knowledge efficiently with videos, and then conduct in-depth exploration and transfer application with VR; Or, before or after the VR experience, video explanations can be used to sort out and consolidate knowledge. For schools lacking large-scale VR devices, choosing immersive videos recorded from the first-person perspective of VR may be more effective than traditional videos, as it can simulate the spatial sense of VR to a certain extent. (5) Attach importance to and adopt multiple methods to evaluate and cultivate learners’ mental models, especially in VR environments. Encourage learners to take advantage of the visualization and interaction features of VR to actively construct and express, in order to deepen understanding and promote model improvement.

In conclusion, this study deepened the understanding of the adaptation effect between the environment and task difficulty in VR and multimedia video learning environments, providing important theoretical basis and practical guidance for optimizing immersive learning design and enhancing learning outcomes. Future research requires continuous efforts in aspects such as more refined task design, broader disciplinary background, deeper mechanism exploration, and more intelligent technical support.

## Data Availability

The original contributions presented in the study are included in the article/supplementary material, further inquiries can be directed to the corresponding author.
